# Antiviral activity of *Scutellaria baicalensis Georgi Extract* against Getah virus *in vivo* and *in vitro*

**DOI:** 10.3389/fvets.2025.1551501

**Published:** 2025-03-25

**Authors:** Baoling Liu, Yuling Wang, Lina Shao, Yuanhang Chen, Zhiwen Xu, Ling Zhu

**Affiliations:** ^1^College of Veterinary Medicine, Sichuan Agricultural University, Chengdu, China; ^2^Sichuan Key Laboratory of Animal Epidemic Disease and Human Health, Sichuan Agricultural University, Chengdu, China

**Keywords:** antiviral, *Scutellaria baicalensis Georgi Extract*, Getah virus, natural compounds, traditional Chinese medicine

## Abstract

**Introduction:**

The Getah virus (GETV) is a zoonotic arbovirus causing disease in humans and animals, a member of the *Alphavirus* genus. Currently, approved antiviral drugs and vaccines against alphaviruses are few available. This study aimed to investigate the anti-GETV activity of the Extract of *Scutellaria baicalensis Georgi* (ESG) *in vivo* and *in vitro*.

**Methods:**

The cytotoxic effects of ESG on BHK-21 cells were quantitatively evaluated through the MTT assay. Quantitative analysis of viral replication was performed using qRT-PCR, while E2 protein expression was analyzed through western blotting. Furthermore, molecular docking simulations were conducted to examine the binding affinity between the principal bioactive constituents of ESG and the E2 structural proteins. Additionally, the therapeutic potential of ESG in alleviating viremia was evaluated in GETV-infected mouse models.

**Results:**

The results showed that ESG significantly attenuated the cytopathic effects induced by GETV infection in BHK-21 cells, concurrently reducing both viral replication and E2 protein expression. Notably, ESG exhibited its most potent antiviral activity during the viral attachment and entry phases, with IC50 values of 3.69 μg/mL and 3.94 μg/mL, respectively. At a concentration of 10 μg/mL, ESG achieved 95.08% inhibition efficiency against viral attachment. Furthermore, in vivo studies revealed that ESG treatment significantly reduced the peak viral load and shortened the duration of viremia in GETV-infected mice. The main components of ESG are baicalin and baicalein, and molecular docking simulations demonstrated strong binding affinities between these compounds and the active site of GETV E2 protein, with docking scores of −6.99 kcal/mol for baicalin and −5.21 kcal/mol for baicalein.

**Conclusion:**

The experimental findings demonstrate that ESG exhibits significant antiviral efficacy against GETV infection both *in vitro* and *in vivo*. These results indicate that ESG represents a promising therapeutic candidate for the prevention and treatment of GETV infections. Mechanistically, the antiviral activity of ESG appears to be mediated, at least in part, through the modulation of E2 protein expression.

## 1 Introduction

Getah virus (GETV) belongs to the Alphavirus genus in the Togaviridae family and is a zoonotic arbovirus causing disease in humans and animals (1, 2). GETV has a broad host range, such as monkeys, birds, pigs, horses, and other mammals that may be infected, which can cause pyrexia and reproductive losses in animals. A 2015 outbreak of GETV infection occurred among Japanese racehorses sequentially to an outbreak in 2014 at the same site (3). In 2017, an outbreak of GETV infection among pigs in China, and more than 150 pregnant sows had stillbirths or fetal mummies in this outbreak (4). GETV has brought a greater risk to the animal husbandry industry and constitutes a massive threat to public health. However, approved antiviral drugs and vaccines against alphaviruses are few available (5, 6). Currently, there are no specific treatments or drugs available for GETV infection (7). Because GETV still represents a significant threat to human and animal health worldwide, developing effective weapons against GETV remains a top priority.

Traditional Chinese Medicine (TCM) is a holistic system of healthcare that has been practiced for thousands of years. *Scutellaria baicalensis Georgi*, as one of the TCMs is a widely distributed plant has been used for heat-clearing and detoxifying. The main active components in *Scutellaria baicalensis Georgi* are flavonoids, terpenoids, and volatile oils. baicalein, baicalin, and wogonin are the essential active substances in the Extracts of *Scutellaria baicalensis Georgi* (ESG) (8). Among them, baicalin has a variety of biological functions, such as anti-bacterial, anti-inflammatory, antiviral, and anti-tumor activities (9). Relevant studies have shown that baicalin inhibits avian infectious bronchitis virus (IBV) and Porcine reproductive and respiratory syndrome virus (PRRSV) (10, 11). The findings of Moghaddam et al. showed that baicalin as the main metabolite of baicalein exerts against dengue virus activity *in vitro* (12). NIU et al. proved that baicalin effectively inhibits PRV infection (13). Although the antiviral activity of baicalin has been reported, the effect of baicalin or ESG against GETV infection remains to be investigated.

This study aimed to investigate the anti-GETV activity of the ESG *in vivo* and *in vitro*, to provide a scientific foundation for preventing and controlling GETV.

## 2 Materials and methods

### 2.1 Cell, virus, and drugs

BHK-21 cells were cultured and passaged at 37°C and 5% CO_2_ and maintained in DMEM (gibco) supplemented with 10% fetal bovine serum (FBS) and antibiotic/antimycotic solution. In this study GETV SC201807 strain (GenBank number: MK693225) was used, and the virus was maintained at Sichuan Agricultural University, College of Veterinary Medicine.

Extracts of Scutellaria baicalensis Georgi (ESG, Baicalin HPLC >85%) were purchased from Baoji Fangsheng Biological Development Co., Ltd (Baoji, China). Dissolve it to 40 mg/mL with DMSO, freeze it at −20°C as stock solution and dilute to working concentration with DMEM when using.

### 2.2 Mice and reagents

All mice were kept in a pathogen-free environment and fed ad-lib. The procedures for care and use of animals were approved by the Ethics Committee of the Veterinary Medicine College of Sichuan Agricultural University, and all applicable institutional and governmental regulations concerning the ethical use of animals were followed.

Mouse Polyclonal Antibody to GETV E2 protein deposited in Animal Biotechnology Center, Sichuan Agricultural University, China. FITC-goat anti-mouse IgG fluorescent secondary antibody was purchased from Sangon Biotech (Shanghai) Co., Ltd (Shanghai, China).

### 2.3 Effect of ESG on the BHK-21 cell viability assay

The cytotoxicity of ESG on the viability of BHK-21 cells was determined using the MTT assay. The BHK-21 cells were cultured to a monolayer with a 96-well cell culture plate. The cells were treated with the ESG diluted by DMEM supplemented with 2% FBS in seven concentrations of 40, 20, 10, 5, 2.5, 1.25, and 0.625 μg/mL, and incubated at 37°C with 5% CO_2_ for 48 h. Each concentration was repeated in four wells, and 0.1% DMSO and cell control were set. After 48 h, MTT solution was added to each well and the microplate was kept at 37°C for 4 h in a humidified atmosphere with 5% CO_2_. Then, solubilization/stop solution was added to the wells, and the absorbance values of the wells were measured at 570 nm using a 96-well plate reader. Drug concentrations with cell viability more significant than 90% were taken as the maximum non-toxic dose (MNTD).

### 2.4 Determination of anti-GETV activity of ESG

A continuous treatment assay was conducted to determine the antiviral activity of ESG against any stage of the GETV life cycle. According to the method Moghaddam et al. proposed (12). BHK-21 cells were cultured to a monolayer in a 96-well cell culture plate. Then the cells were treated with low (2 μg/mL), medium (6 μg/mL), and high (10 μg/mL) doses of the ESG, and the supernatant was discarded after incubation for 5 h. The mixture of ESG and GETV (MOI = 0.1) was added to the culture plate, and the supernatant was discarded after 1 h incubation. The supernatant was washed with PBS three times, and the corresponding concentration of drug solution was added to each well. At the same time, virus control, cell control, and ribavirin (10 μg/mL) control groups were set. After incubation at 37°C with 5% CO_2_ for 48 h, cytopathic effect (CPE) was observed, MTT analysis was performed, and the supernatant was collected for the following assay.

### 2.5 Quantitative reverse transcription-polymerase chain reaction (qRT-PCR) was used to detect the effect of ESG on virus replication

The GETV yield during the antiviral studies was evaluated by qRT-PCR assay. A total of 300 μL of supernatant collected in the previous step was taken, and the viral RNA was extracted using Trizol (Invitrogen, Beijing, China), and the cDNA was generated. Then the amplification of the E2 encoding sequence was performed using the primers E2-F (5′-CTTGACGGTAAGGTCACGGG-3′) and E2-R (5′-GTAAGCTTCGCTAGGTCGGG-3′). The cycling parameters were 94°C for 180 s, 35 cycles of 95°C for 10 s, and 58°C for 15 s. The amplified product was verified by melting curve analysis at 58°C.

### 2.6 Western blotting analysis

Western Blotting (WB) assay was used to verify further the inhibitory effect of the ESG on E2 protein expression of the GETV virus. Total proteins were extracted from GETV-infected cells with protein extraction reagent (Beyotime, China) according to the manufacturer's instructions. The protein concentration was measured using the BCA kit (Thermo, USA). After being isolated using 12% SDS-PAGE, the proteins were transferred to a nitrocellulose membrane. The mouse polyclonal antibodies against GETV E2 protein (1:100) and GAPDH (1:5000) were incubated as primary antibodies, and the secondary antibodies were diluted at 1:3000 and incubated at 37°C for 1 h. Imaging was performed with the hyper signal detection of protein bands by the West Pico PLUS chemiluminescence Branch and ChemiDoc MP Bio-Rad chemiluminescence imaging system. Image J software analyzed optical density values, and changes in E2 protein expression were compared.

### 2.7 Time-of-addition assay

**Figure 1:**
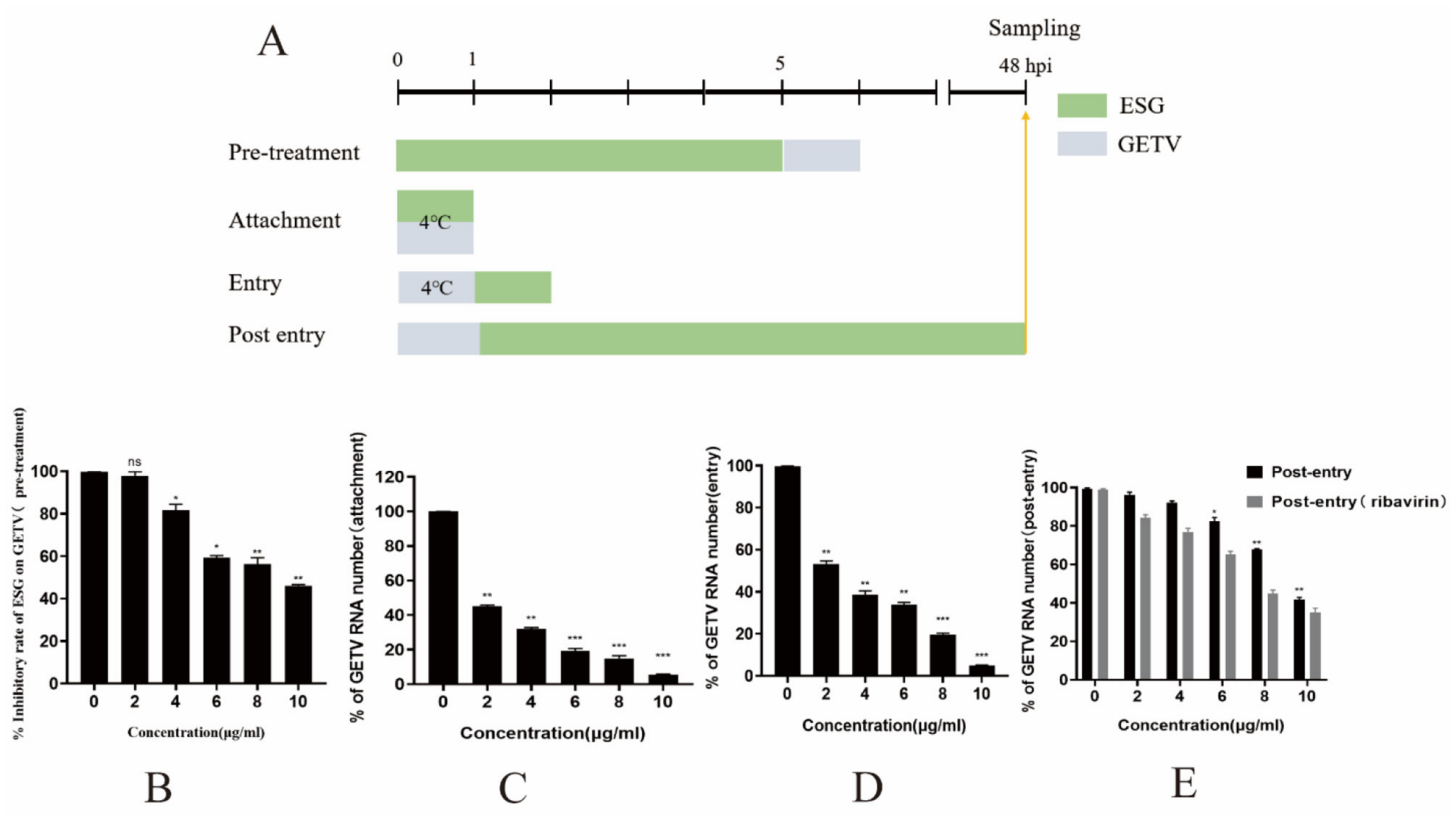
ESG inhibits GETV replication in four treatment modes. **(A)** PRRSV (0.1 MOI) was used to infect the cells for 1 h for all four treatment modes. Cells and supernatant were collected at 48 h. Except for the temperatures specifically labeled at specific times in the figure, all treatment temperatures were at 37°C. **(B–E)** GETV mRNA levels in four treatment modes. The difference was considered statistically significant when *P* < 0.05 (^***^P < 0.001; ^**^P < 0.01; ^*^P < 0.05). Non-linear regression analysis was done using GraphPad Prism version 8 software.

To analyze the effect of ESG on GETV, experiments were conducted with reference to previous research methods (10, 14, 15). BHK-21 cells were cultured to a monolayer with a 24-well plate, 2, 4, 6, 8, and 10 μg/mL ESG was added before (pre-treatment), during (attachment treatment), or after (entry and post-entry treatment) GETV infection (Figure 1A). For pretreatment BHK-21 cells were incubated with ESG at 37°C for 5 h, washed three times with PBS and infected with GETV (0.1 MOI) at 37°C for 1 h. For attachment treatment BHK-21 cells were incubated with ESG and GETV at 4°C for 1 h. For entry BHK-21 cells were infected with GETV (0.1 MOI) at 4°C for 1 h, washed three times with PBS and incubated with ESG at 37°C with for 1 h. For post entry treatment BHK-21 cells were infected with GETV (0.1 MOI) at 37°C for 1 h, washed three times with PBS and incubated with ESG at 37°C with 5% CO_2_ until 48 h. Since the antiviral activity of ribavirin is intracellular or mainly in RNA replication, it has been used only to control this antiviral assay. All four treatment modalities were incubated in a CO_2_ incubator at 37°C for up to 48 h after treatment. Cells and supernatant were collected at 48 h post-infection (hpi). Detection of virus multiplication by qRT-PCR.

### 2.8 Effect on viremia in mice infected with GETV

For this assay, 120 three-week-old male mice were randomly divided into four groups: A, B, C, and D. The existing GETV infection model in our laboratory showed that the LD50 of the SC201807 strain in mice is 10^2.85^ TCID50. Mice in groups A, B, and C were intranasally inoculated with 4 × LD50 (4 × 10^2.85^ TCID50) of GETV on the 3rd day of the study. Mice in group A were given ESG 3 days before GETV infection at a dose of 3 mg/mouse/day; mice in group B were given ESG on the same day as GETV; mice in group C were only infected with GETV without being given drugs; mice in group D were the negative control group, and each group was treated with individual feeding and management. The clinical symptoms of each group of mice were observed daily, and blood was collected from five mice in each group at 24 h, 48 h, 72 h, 7 days, 14 days, and 21 days after GETV infection, followed by GETV viremia testing. Table 1 shows the animal grouping and treatment plan.

**Table 1:** Grouping of experimental animals and treatment plan.

**Treatment group**	**A**	**B**	**C**	**D**
Number of mice	30	30	30	30
Dosage per mouse (mg/day)	3	3	_	_
Start time of treatment	−3	0	_	_
Way of infection	Vaccination of 4 × LD50 doses of GETV solution intranasally			

### 2.9 Molecular docking

To investigate the interaction effects between baicalein and baicalin—the primary active components of ESG—and the E2 protein, predictive structural modeling of the E2 protein was conducted using AlphaFold 3. Subsequently, molecular docking analyses were performed to examine the binding interactions of the E2 protein with baicalein and baicalin individually, following the methodology described by Chakraborty et al. (16).

### 2.10 Statistical analysis

One-way analysis of variance (ANOVA) was conducted with the Graph Pad Prism 5 software; *P* < 0.05 was considered a statistically significant difference.

## 3 Results

### 3.1 Cytotoxicity of ESG on BHK-21

**Figure 2:**
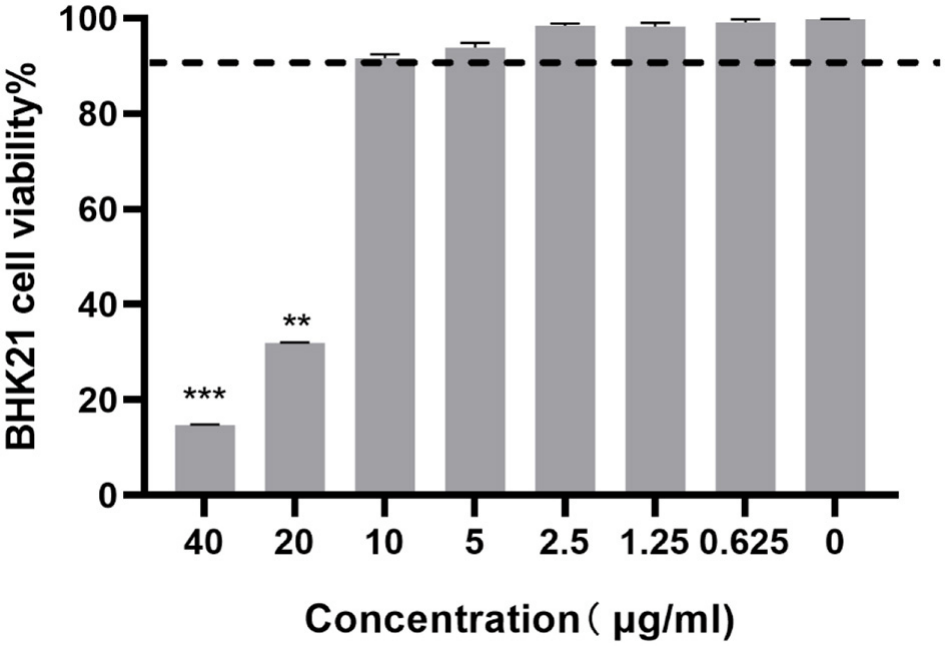
The cytotoxicity of ESG on BHK-21 cells by the MTT assay. The difference was considered statistically significant when *P* < 0.05 (^***^P < 0.001; ^**^P < 0.01). Non-linear regression analysis was done using GraphPad Prism version 8 software.

Cytotoxicity assay was performed to determine the non-toxic concentrations of ESG against BHK-21 cells using the MTS assay. The cells treated with 0.1% DMSO showed no cytotoxicity. The BHK-21 cell viability decreased when the drug concentration increased. At a concentration of 10 μg/mL, the viability of ESG-treated BHK-21 cells was >90% compared to vehicle control indicating that the concentration could be considered as the maximum non-toxic dose (MNTD) of ESG for BHK-21 cells (Figure 2). Therefore, 2, 4, 6, 8, and 10 μg/mL were finally selected as the working concentrations for follow-up experiments.

### 3.2 ESG reduces the effects of GETV on CPE and viability

**Figure 3:**
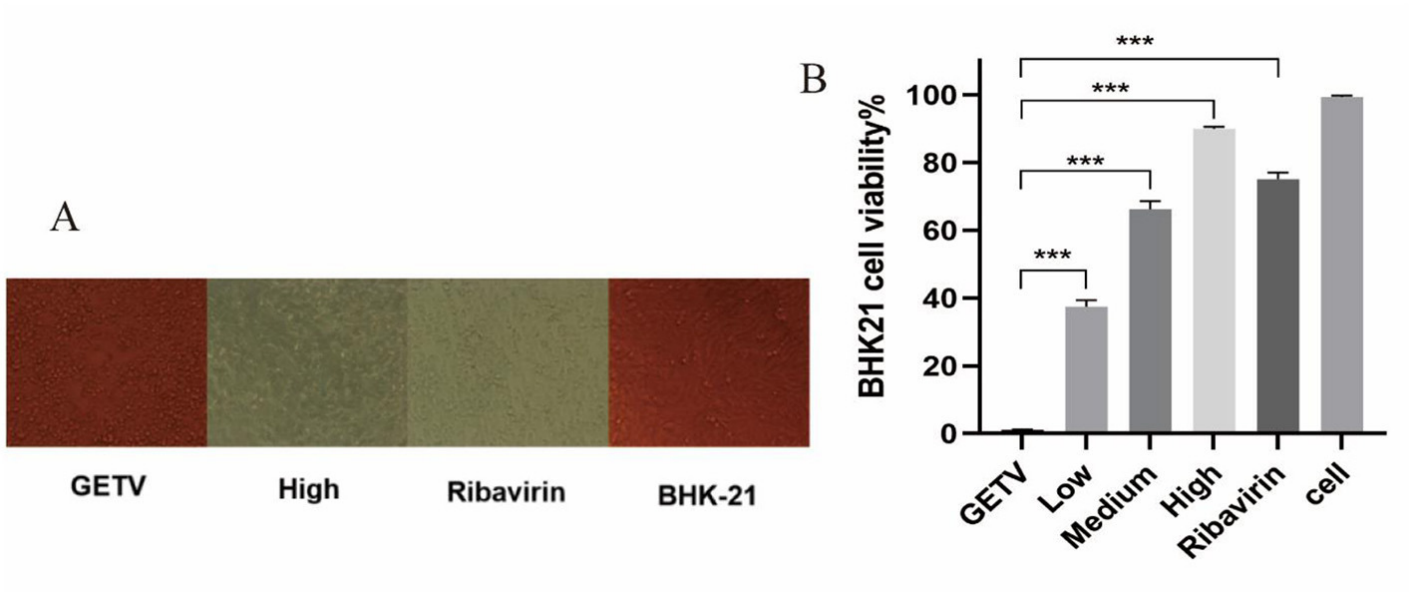
Inhibitory effects of ESG on the GETV. The difference was considered statistically significant when *P* < 0.05 (^***^P < 0.001). Non-linear regression analysis was done using GraphPad Prism version 8 software. **(A)** Effect of ESG on the morphology of BHK-21 cells. **(B)** Survival rate of BHK-21 cells measured by MTT.

To identify whether the ESG has antiviral effects, different drug concentrations were tested on GETV-infected BHK-21 cells. Observing the CPE condition under a microscope, the results showed that the cell particles in the GETV control group increased; they were rounded, and most of them fell off, with an apparent degree of CPE lesions. Compared with the virus control group, the cells in the high-dose treatment group and the ribavirin control group were similar, most of which were in an adhesive state, and the cell morphology was normal (Figure 3A). Regarding the MTT experiment and the cell survival rate, after GETV infection, the cell survival rate decreased significantly; compared with the virus infection control group, the cell viability of the groups receiving high, medium, and low doses of *Scutellaria baicalensis* extract and of the ribavirin group was significantly increased (Figure 3B), indicating that the extract significantly inhibited the pathological effect of BHK-21 cells caused by GETV.

### 3.3 ESG can significantly inhibit viral RNA replication

**Figure 4:**
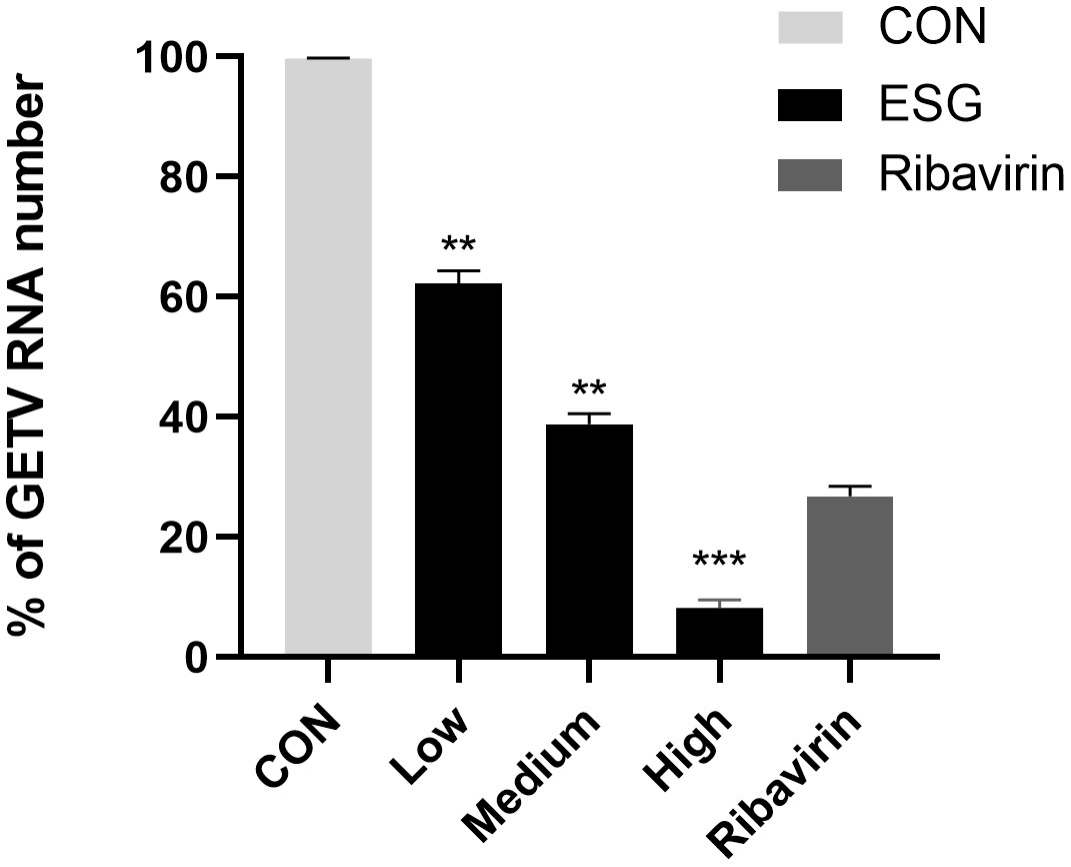
Effect of high, medium and low doses of ESG on GETV virus replication. The difference was considered statistically significant when *P* < 0.05 (^***^P < 0.001; ^**^P < 0.01). Non-linear regression analysis was done using GraphPad Prism version 8 software.

The results of the qRT-PCR showed that high, medium and low doses of ESG could inhibit viral RNA replication; an effective reduction in virus copy number could still be achieved at a drug concentration as low as 2 μg/mL (Figure 4).

### 3.4 ESG reduces the expression of E2 protein

**Figure 5:**
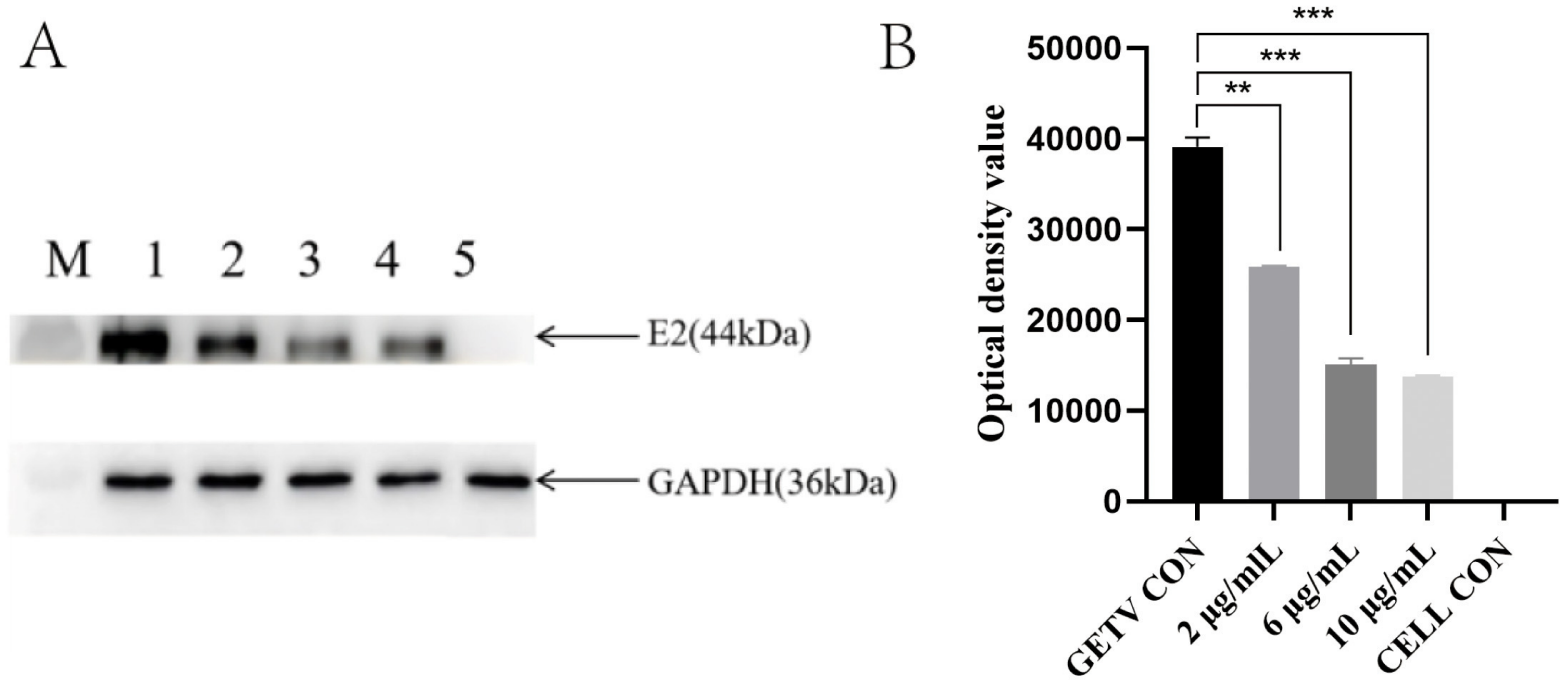
Effects of the high, medium, and low doses of ESG on GETV virus replication. **(A)** Western blot result; M, Marker; 1; GETV control; 2–4; Treat with 2, 6, 10 μg/mL ESG; 5; BHK-21 cell control. **(B)** Optical density value statistics chart. The difference was considered statistically significant when *P* < 0.05 (^***^P < 0.001; ^**^P < 0.01). Non-linear regression analysis was done using GraphPad Prism version 8 software.

Western blotting was used to determine whether ESG had an inhibitory effect on the expression of E2 protein. The WB test results showed that compared with the virus control group, as the concentration of ESG increased, the GETV E2 protein expression gradually decreased. When the drug concentration reached 10 μg/mL, the band's color was lighter. The inhibitory effect was more evident (Figure 5A). This indicates that ESG significantly inhibited the expression of E2 protein in BHK-21 cells. The optical density statistics showed that ESG significantly reduced the expression of E2 protein (Figure 5B). In this step, GAPDH was used as an internal reference to eliminate the cells' interference with the experimental results.

### 3.5 ESG can effectively inhibit the virus in the attachment and entry stages

According to the results of the ratio of viral gene copy numbers in the experimental group and the control group, ESG had the most potent inhibitory effect on the GETV attachment stage (Figure 1C). When BHK-21 cells were pretreated with ESG for 5 h and then inoculated, the inhibitory effect on GETV infection was not noticeable (IC50 = 8.63 μg/mL). When the concentration was 10 μg/mL, the inhibition rate was only 53.86%. At different doses, ESG has a significant inhibitory effect on the attachment of GETV (IC50 = 3.59 μg/mL) in a dose-dependent manner. It could still be effectively inhibited when 2 μg/mL of the drug concentration. An inhibition rate reached 49.48%; when the drug concentration was 10 μg/mL, the inhibition rate advanced to 94.49%. These results indicate that the ESG has an inhibitory effect on the replication of GETV after GETV enters the cell in a dose-dependent manner. However, the drug's inhibitory effect after the virus had entered the cell (IC50 = 9.4 μg/mL) was lower than that of ribavirin (IC50 = 7.74 μg/mL) (Figure 1E). When the concentration of ESG was 10 μg/mL, the inhibition rate of the virus was still below 50%.

### 3.6 ESG can effectively relieve viremia in mice

**Figure 6:**
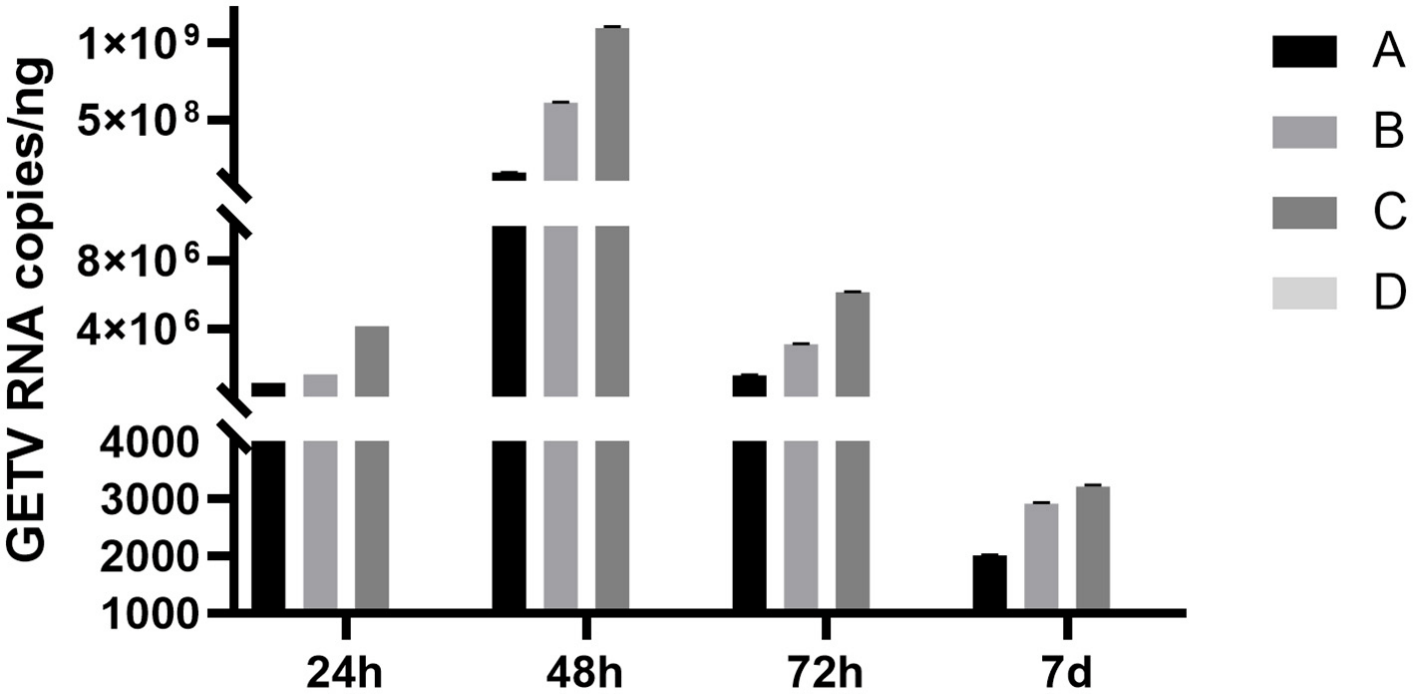
Changes in the copy numbers of the GETV in the blood of mice at different times after infection. A, B, and C denote groups A, B, and C, respectively. Mice in group A were given ESG 3 days before GETV infection at a dose of 3 mg/mouse/day; mice in group B were given ESG on the same day as GETV; mice in group C were only infected with GETV without being given drugs; mice in group D were the negative control group, and each group was treated with individual feeding and management.

The RT-qPCR method was used to analyze the whole blood samples of mice at 24 h, 48 h, 72 h, 7 days, 14 days, and 21 days after inoculation. Blood from mice in groups A, B, and C was positive for GETC from 24 h. The positivity rate in group A was 5/5 on the 7th day and decreased to 0/5 on the 14th day, whereas group B was 1/5 on the 14th day and dropped to 0/5 on the 21st day. The rate in group C was 3/5 on the 14th day and fell to 0/5 on the 21st day, whereas group D was 0/5 (Table 2). Some mice in groups A and B were negative for GETV on the 7 and 21 days. Therefore, quantitative analysis of the GETV content in the whole blood of mice was only performed at the 12th h, 24th h, 48th h, 72th h, and 7th day (Figure 6). The virus copy number of mice in groups A, B, and C peaked at 48 h after infection and gradually decreased. The viral copy numbers in group A and group B were significantly lower than those in group C at 48 h after the virus challenge (*P* < 0.05; Figure 6).

**Table 2:** GETV positive rate in mice blood.

**Period**	**Positive rate**			
	**Group A**	**Group B**	**Group C**	**Group D**
24 h	5/5	5/5	5/5	0/5
48 h	5/5	5/5	5/5	0/5
72 h	5/5	5/5	5/5	0/5
7 day	5/5	5/5	5/5	0/5
14 day	0/5	1/5	3/5	0/5
21 day	0/5	0/5	0/5	0/5

### 3.7 Molecular docking

**Figure 7:**
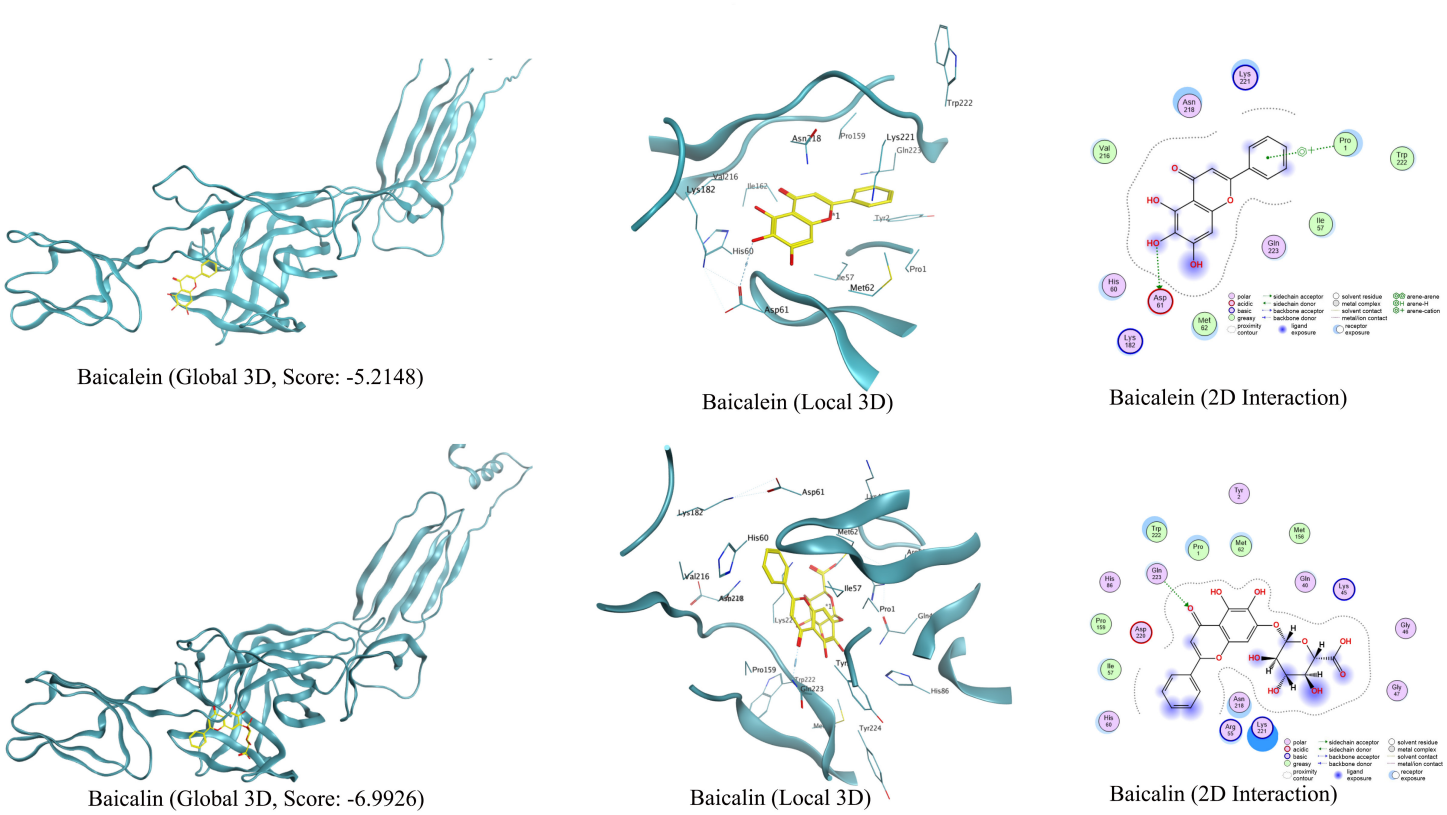
Viral target E2 protein docked with baicalin and baicalein, and their interactive residues at the active sites are shown in the images.

The molecular docking results showed that baicalin and baicalein bound to the active site of the E2 protein of GETV with docking scores of −6.99 and −5.21 kcal/mol (Figure 7), respectively, which demonstrated strong binding affinities between these compounds and the active site of GETV E2 protein. The inhibition of GETV by ESG may be through the regulation of the expression of E2 protein.

## 4 Discussion

GETV threatens public health continuously because it can infect humans and many animals. Clinically, GETV can cause fever, diarrhea, and reproductive disorders in pigs, representing significant threats to pig breeding (17). Currently, there are no effective drugs or vaccines for GETV. Therefore, it is still a challenge to prevent GETV infection and it is necessary to develop some new effective antiviral strategies or drugs to combat GETV infection.

In this study, we evaluated ESG anti-GETV activity *in vivo* and *in vitro*. We found that the mixed inoculation of ESG and GETV could effectively inhibit the virus infection, and the IC50 of GETV was 3.59 and 3.94 μg/mL, respectively. When the concentration of ESG in the adsorption process reached 10 μg/mL, it inhibited 95.08% of the GETV replication efficiency. Still, the pre-treatment and the post-entry stage inhibitory effects were not noticeable. Therefore, it is believed that ESG plays a role in the early stages of GETV infection. We suggest that ESG may act directly on the virus instead of stimulating the immune response of host cells to achieve the effect of viral suppression.

The main structural proteins of the Getah virus are Cap, E3, E2, 6K, and E1 proteins. Research on antiviral strategies against alphaviruses usually focused on the E1 and E2 proteins, which bind to cells during the endocytosis of the virus (18). In addition, E2 is also necessary for GETV budding. Western blot results showed that ESG inhibited the expression of E2 protein. The detection of E2 showed that GETV replicates and assembles successfully in the cell; therefore, ESG can also play a role in the assembly and release process of the virus in the later stages of the virus entering the cell. The results of molecular docking further confirmed that baicalin and baicalein, the main components of ESG, can bind to E2 protein, proving that E2 protein is an important target.

GETV and Chikungunya virus (CHIKV) belong to the genus Alphavirus of the Togaviridae, and ZIKV and DENV belong to the Flaviviridae. They have similar structures and are both enveloped RNA viruses of the arboviruses. Studies have shown that baicalein can inhibit the attachment of CHIKV on host cells and has a high inhibitory effect on the RNA replication process after ZIKV enters the cell; it can also significantly inhibit the ZIKV from entering the cell (15, 19). Hassandarvish et al. have shown that baicalein and baicalin affect the virus replication cycle by interacting with Dengue virus (DENV) essential proteins, such as E, NS2B-NS3, and NS5 (20). The results of this experiment are consistent with these conclusions. The similar antiviral activity of baicalein against these viruses indicates that they may act on the same mechanism.

The antiviral experiment using ESG was carried out in mice, and ESG could effectively reduce viremia in the mouse model. It could significantly reduce the GETV load in the body within 14 days after infection and accelerate the removal of GETV. In addition, the effect of the early preventive medication is better than that of simultaneous medicines, which may be related to the particular blood concentration of the mice before GETV infection. It is suggested that ESG can be used to prevent and treat GETV. In addition, ESG can shorten the duration of GETV viremia. Our results lead us to infer that to reduce the time for the vaccine strain to carry the virus and reduce the risk of attenuated vaccine mutation returning to strong, ESG can be used after inoculation with GETV-attenuated vaccine to facilitate the later development and application of Getah virus vaccine.

This study shows that the ESG can inhibit the attachment and entry of the virus into the cells *in vitro*, thereby inhibiting its infection of host cells. For the first time, the results show that the ESG can reduce the peak value of GETV viremia and the duration of low-level viremia in mice, and contribute to reducing the risk of virus transmission, which provides a specific reference for the prevention and control of GETV.

## Data Availability

The datasets presented in this study can be found in online repositories. The names of the repository/repositories and accession number(s) can be found in the article/supplementary material.
